# Management of anorexia and bulimia nervosa: An evidence-based review

**DOI:** 10.4103/0019-5545.64596

**Published:** 2010

**Authors:** Kaustav Chakraborty, Debasish Basu

**Affiliations:** Department of Psychiatry, Postgraduate Institute of Medical Education and Research, Chandigarh -160 012, India

**Keywords:** Anorexia nervosa, behavioural interventions, bulimia, eating disorder, management, medications, psychotherapy

## Abstract

Anorexia nervosa and bulimia nervosa are primarily psychiatric disorders characterized by severe disturbances of eating behavior. Eating disorders are most prevalent in the Western culture where food is in abundance and female attractiveness is equated with thinness. Eating disorders are rare in countries like India. Despite a plethora of management options available to the mental health professionals, no major breakthrough has been achieved in recent years. Nutritional rehabilitation along with some form of re educative psychotherapy remains the mainstay of management of anorexia nervosa. In bulimia nervosa, both fluoxetine and cognitive behavior therapy have been found to be effective. Although the above-mentioned management options have been in use for decades, the active ingredient is still to be ascertained.

## INTRODUCTION

Eating disorder is defined as a persistent disturbance of eating behavior or behavior intended to control weight, which significantly impairs physical health or psychosocial functioning.[1] Anorexia nervosa (AN) is a type of eating disorder marked by an inability to maintain a normal healthy body weight, often dropping below 85% of ideal body weight (IBW). Bulimia nervosa (BN) is characterized by recurrent episodes of binge eating in combination with some form of inappropriate compensatory behavior.

In young females in Western Europe and the United States, the mean prevalence estimates are 0.3% for AN, 1.0% for BN; sub threshold conditions of clinical concern are more prevalent.[2] Eating disorders are chronic psychiatric conditions. Although some patients with AN improve symptomatically over time, a substantial proportion continues to have body image disturbances, disordered eating, and other psychiatric difficulties.[3–5] A review of a large number of studies of patients of AN who were hospitalized or who received tertiary-level care and were followed up at least four years after the onset of illness indicates that "good" outcomes occurred in 44% of the patients and approximately 5% of the patients died.[3] In case of BN, the overall short term success rate for patients receiving psychosocial treatment or medication has been reported to be 50 - 70%.[4] Relapse rates of 30 - 85% have been reported for successfully treated patients at six months to six years of follow-up.[6,7]

Although widely described in Western literature, anorexia nervosa and related eating disorders are rare in non-western cultures. In India, the information regarding these disorders is very limited.[8] Indian patients chiefly present with refusal to eat, persistent vomiting, marked weight loss, amenorrhea and other somatic symptoms, but do not show over activity or disturbances in body image seen characteristically in anorexia nervosa.[9]

Mortality rates in eating disorders, specifically anorexia nervosa, are among the highest in the mental disorders.[3,7,10] The scenario does not appear to have improved during the 20th century despite the plethora of options available to the psychiatrists as very few patients utilize the healthcare facilities.[11]

Thus it becomes prudent to review the management of eating disorder to have a better understanding of this puzzling topic. For this purpose the wealth of evidence has been subdivided under two broad categories namely - anorexia nervosa and bulimia nervosa.

### Data search methodology

The data search strategies for this review included electronic databases as well as hand-search of relevant publications or cross-references. The electronic search included PUBMED and other search engines (e.g. Google Scholar, PsychINFO). Cross-searches of electronic and hand search key references often yielded other relevant material. The search terms used, in various combinations, were: anorexia, bulimia, treatment, management, medications, behavioral interventions, psychosocial interventions.

### Data review methodology

The research/data inclusion for this review was dictated by the following principles. We included studies conducted after 1990. We were deliberately over-inclusive and liberal in our approach and did not stick to any standardized methodology for data inclusion. Rather we tried to include as much research as possible to cover as many aspects of the research on the treatment of anorexia and bulimia nervosa. Wherever applicable, the strengths and the limitations of the research being cited were discussed alongside the description of the research.

## GENERAL PRINCIPLES IN THE MANAGEMENT OF EATING DISORDERS

To establish and maintain a therapeutic alliance is of utmost importance in the management of eating disorders. Many patients with anorexia nervosa are initially reluctant to enter treatment and may remain preoccupied with their symptoms. Many are secretive and may withhold information about their behavior because of shame. Encouraging patients to gain weight could generate extreme anxiety in them. Addressing patients’ resistance to treatment and enhancing their motivation for change is an important aspect of management of eating disorders.[12–15]

Management of eating disorders should be a multidisciplinary approach involving psychiatrists, psychologists, endocrinologists, dentists, gastro enterologists, internists so on and so forth. All personnel must work closely together and maintain open communication and mutual respect.

### Assessment of eating disorder symptoms

An assessment of eating disorder symptoms will assist the clinician in identifying target symptoms and behaviors that will be addressed in the treatment plan as well as in determining the diagnosis of eating disorder. A detailed report of food intake during a single day in the patient’s life may be quite informative. A family history should be obtained regarding eating disorders and other psychiatric disorders, alcohol and other substance use disorders, obesity, family interactions in relation to the patient’s disorder, and family attitudes toward eating, exercise, and appearance.

There are instruments which can be used by clinicians to interview patients in a structured format and can be regarded as "gold standards" to determine clinical diagnoses. There are clinician administered measures like Eating Disorder Examination (EDE) and Yale-Brown-Cornell Eating Disorder Scale (YBC-EDS), which can be completed within 10-40 minutes.[16–18] Self reported instruments e.g. Diagnostic Survey for Eating Disorders (DSED), Bulimia Test-Revised (BULIT-R), Eating Attitudes Test (EAT), Eating Disorder Examination-Questionnaire (EDE-Q), Eating Disorders Inventory-2 (EDI-2), Eating Disorders Questionnaire (EDQ) etc. are also helpful for initial screening purpose but should be supplemented by detailed assessment by trained clinical interviewers.[19–24]

### Assessment of patient’s physical status

A detailed physical examination should be conducted by a physician familiar with common findings in patients with eating disorders, with particular attention to vital signs; physical status (including height and weight); heart rate and rhythm; jugular venous pressure; heart sounds (especially midsystolic clicks or murmurs from mitral valve prolapse); acrocyanosis; delayed capillary refill; lanugo; salivary gland enlargement; scarring on the dorsum of the hands (Russell’s sign); evidence of self-injurious behavior such as ecchymoses, linear scars, and cigarette burns; muscular weakness; indications of muscular irritability due to hypocalcaemia, such as in Chvostek’s and Trousseau’s signs; and gait and eye abnormalities.[25–27] BMI, in conjunction with weight and height, has gained increasing attention in research and clinical settings as a tool for assessing eating disorder patients. Regular monitoring of BMI should be done. There are a host of other biochemical abnormalities to which attention has to be paid and those should be adequately treated.

### Choice of treatment setting

Patients of eating disorders can be treated in intensive inpatient settings (in which subspecialty general medical consultation is readily available) to residential and partial hospitalization programs to varying levels of outpatient care. Patients who weigh less than approximately 85% of their individually estimated healthy weights have considerable difficulty gaining weight outside of a highly structured program. Healthy weight estimates for a given individual must be determined by that person’s physicians on the basis of historical data (e.g., growth charts) and, for women, the weight at which healthy menstruation and ovulation resume, which may be higher than the weight at which menstruation and ovulation became impaired.[28–30]

Others factors which determine hospitalization include a rapid or persistent decline in oral intake; a decline in weight despite maximally intensive outpatient or partial hospitalization intervention; the presence of additional stressors, such as dental procedures, that may interfere with the patient’s ability to eat; the weight at which the patient was medically unstable in the past; co-occurring psychiatric problems that merit hospitalization; and patient’s denial and resistance to participate in his or her own care in less supervised settings.[31]

## TREATMENT OF ANOREXIA NERVOSA

### 1. Nutritional rehabilitation

The goals of nutritional rehabilitation for seriously underweight patients are to restore weight, normalize eating patterns, achieve normal perceptions of hunger and satiety, and correct biological and psychological sequelae of malnutrition.[32,33] A healthy goal weight for female patients is the weight at which normal menstruation and ovulation are restored and, for male patients, the weight at which normal testicular function is resumed.[34] In one study of 100 adolescent patients with anorexia nervosa, the resumption of menses occurred at a weight approximately 4.5 pounds greater than the weight at which menses was lost; at 90% of healthy weight, 86% of patients resumed menses.[35]

Refeeding programs should be implemented in nurturing emotional contexts. Nursing supervised oral refeeding of normal food in appropriate amounts and composition is preferred. Clinical consensus suggests that realistic targets are 2-3 lb/week for hospitalized patients and 0.5-1 lb/week for individuals in outpatient programs, although an intensive partial hospitalization, stepped-down program has reported gains of up to 2 lb/week.[36] Occasionally, some patients may gain as much as 4-5 lb/week, but these individuals must be carefully monitored for refeeding syndrome and fluid retention. Intake levels should usually start at 30-40 kcal/kg per day (approximately 1,000-1,600 kcal/day). During the weight gain phase, intake may have to be advanced progressively to as high as 70-100 kcal/kg per day for some patients; many male patients require a very large number of calories to gain weight.[34]

Forced nasogastric or parenteral feeding can each be accompanied by substantial dangers. When nasogastric feeding is necessary, clinical experience suggests that continuous feeding (i.e., over 24 hours) may be less likely than three to four bolus feedings a day to result in metabolic abnormalities or patient discomfort and may be better tolerated by patients.[34] As an alternative to nasogastric feeding, in very difficult situations where patients physically resist and constantly remove their nasogastric tubes, gastrostomy or jejunostomy tubes may be surgically inserted.[34] Ten to fifteen per cent cases of AN require hospital based involuntary treatment.[37] The general agreement is that children and adolescents who are severely malnourished and in grave medical danger should be refed, involuntarily if necessary, but that every effort should be made to gain their cooperation as cognitive function improves.[38]

For weight maintenance, Kaye *et al*. found that weight-restored patients with anorexia nervosa often require 200-400 calories more than sex-, age-, weight-, and height-matched control subjects to maintain weight.[39] Baran *et al*. assessed weight, height, eating disorder symptoms, and severity of depressive and anxiety symptoms in 22 women with anorexia nervosa at hospital admission and at follow-up occurring a mean of 29 months after patient discharge.[40] The patients who were discharged while severely underweight reported significantly higher rates of rehospitalization and endorsed more symptoms than those who had achieved normal weight before discharge. In a study by Watson *et al*. of 397 patients admitted to an inpatient service over a seven-year period, patients who were admitted involuntarily showed the same short-term rates of weight gain as those who were admitted voluntarily.[41] Moreover, most of those who were involuntarily treated later affirmed the need for and exhibited a better attitude toward the treatment process.

### 2. Medication

#### 2.1. Antidepressants

##### 2.1.1. Selective Serotonin Reuptake Inhibitors

**Fluoxetine:** Two trials used fluoxetine at different stages of refeeding. One inpatient study randomized 31 women (aged 16-45 years) who had achieved weight restoration of at least 65% of ideal body weight (IBW) to fluoxetine (60 mg/day) or placebo.[42] Mean BMI at randomization was 15 kg/m^2^. Patients continued to receive psychotherapy. No significant differences emerged between fluoxetine and placebo on weight gain (16 versus 13 pounds), psychological features of eating disorders, or depression or anxiety measures.

In another study, patients were randomly assigned to either fluoxetine or placebo before inpatient discharge, with a beginning dosage of 20 mg/day adjusted over 52 weeks to a maximum of 60 mg/day.[43] The range of weight for all participants at randomization was 76-100% average body weight (ABW), with the majority above 90%. Outpatient psychotherapy was permitted. At endpoint, patients on medication did not differ significantly from those on placebo on eating, psychological, or biomarker measures.

**Citalopram:** Fassino *et al*. compared 52 adult female patients with anorexia nervosa, restricting type, who received citalopram (*n* = 26) or were assigned to a waiting-list control group (*n* = 26).[44] Although no differences were found in weight gain between the groups, after three months of treatment, those receiving citalopram showed modest advantages regarding symptoms of depression, obsessive-compulsive symptoms, impulsiveness, and trait anger, as assessed by rating scales.

##### 2.1.2. Tricyclic antidepressants

Data on the efficacy of tricyclic antidepressants are even more limited.

Halmi *et al*. compared amitriptyline (160 mg/day), cyproheptadine (32 mg/ day), and placebo in 72 women aged 13-36 years.[45] Daily caloric intake was significantly higher for cyproheptadine than for placebo; significantly fewer days were needed to achieve target weight (in those who did) with both amitriptyline and cyproheptadine groups than with placebo. Attrition was moderately high: amitriptyline group, 30%; cyproheptadine group, 25%; and placebo group, 20%. In another study, amitriptyline in doses up to 175 mg/ day in 25 youth (aged 11-17 years) led to no significant differences in eating, mood, or weight outcomes in comparison with placebo.[46]

In a double-blind, controlled study of 16 patients with anorexia nervosa, by Lacey and Crisp, no significant beneficial effect was observed from adding clomipramine to the usual treatment (although dosages of only 50 mg/day were used).[47]

### Cochrane review of antidepressants in the treatment of anorexia nervosa

A Cochrane review by Claudino *et al*. that included seven studies (four studies compared antidepressants with placebo and other three compared antidepressants) did not find any evidence that antidepressants improve weight gain, eating disorder or associated psychopathology.[48] Isolated findings favoring Amineptine and Nortriptyline emerged from antidepressant versus antidepressant comparison. However, authors concluded that it cannot be viewed as an evidence of efficacy in the light of the findings of the placebo comparisons.

#### 2.2. Antipsychotics

Small open-label studies in adults suggest that low doses of second-generation antipsychotic medications such as olanzapine, quetiapine may improve weight gain and psychological indicators, but controlled studies are needed to confirm this.[49–54]

In an open trial, 13 severely ill outpatients with anorexia nervosa, restricting type received low-dose (1-2 mg) haloperidol in addition to standard treatment and were reported to benefit (significant weight gain and improved insight).[55]

#### 2.3. Hormones

Investigators have studied three hormones in the treatment of AN: growth hormone (rGH), testosterone, and estrogen.

Three weeks of transdermal testosterone (150 mg or 330 mg) administered to 38 patients with AN age of 18 to 50 led to greater decreases in depression in patients who were depressed at baseline, but differences in weight were not interpretable.[56]

Growth hormone (15 mg/kg/day) administered to 14 female patients and one male receiving inpatient care for AN led to fewer days to display normal orthostatic heart rate response to a standing challenge among the treatment group than among placebo group.[57]

Klibanski *et al*. compared estrogen/progesterone (0.625 mg Premarin® or 5 mg Provera® per day) versus nonmedication control in 48 females 16 to 43 years and found no differences between groups on bone density at six months.[58]

#### 2.4. Antiepileptic drugs

A recent review[59] suggested that Carbamazepine and Valproate may be effective in treating patients of anorexia nervosa when they are used to treat an associated psychiatric (e.g. mood) or neurological (e.g. seizure) disorder; otherwise, both agents, particularly valproate, are associated with weight gain.

#### 2.5. Nutritional supplement

Birmingham *et al*. determined that 14 mg per day of zinc, in 54 women inpatients (older than 15 years), was associated with accelerated increase of BMI compared to placebo.[60]

### 3. Psychosocial interventions

Although psychosocial interventions, including psycho education, individual therapy, family therapy and (in some settings) group therapy, are considered to be the mainstay of effective treatment for anorexia nervosa, supporting evidence is sparse.

#### 3.1. Cognitive behavioral therapy (CBT)

CBT studies generally used a form of therapy tailored to anorexia nervosa that focused on cognitive and behavioral features associated with the maintenance of eating pathology. Of the three CBT studies, one followed inpatient weight restoration and two were done in the underweight state.[61–63]

CBT significantly reduced relapse risk and increased the likelihood of good outcome compared to nutritional counseling based on nutritional education and food exchanges after inpatient weight restoration.[61] Of those receiving CBT, a greater number of individuals with good outcomes also received antidepressant medication. One study of underweight AN outpatients compared CBT with IPT and nonspecific supportive clinical management (NSCM).[62] For this study, NSCM reflected the type of treatment an individual could receive in the community from a provider familiar with the treatment of eating disorders and incorporated elements of sound clinical management and supportive psychotherapy. In an intention to - treat analysis, NSCM performed significantly better than IPT in producing global good outcome ratings; CBT outcomes fell in between and were not significantly different from outcomes in the other two treatments.

#### 3.2. Cognitive analytic therapy (CAT)

The two studies that utilized CAT, a treatment which integrates psychodynamic with behavioral factors and focuses on interpersonal and transference issues, failed to find any advantage of CAT over educational behavioral therapy or focal family therapy in eating, mood, or weight outcomes.[64,65]

#### 3.3. Family therapy

Two studies incorporated various forms of family therapy with adults.[65,66] Dare *et al*. found family therapy to be superior to routine treatment but equivalent to a focal time-limited psychodynamic psychotherapy in increasing percentage of adult body weight, restoring menstruation, and decreasing bulimic symptoms; overall clinical improvement was modest, however.[65] Crisp *et al*. found outpatient individual and family therapy with variable numbers of sessions to be superior to referral to a family physician for increased weight at one-and two-year follow-up.[66]

Four family therapy studies focused exclusively on adolescents; and one combined adolescent and adult patients.[67–72] Family therapy was more effective for younger patients with earlier onset than for older patients with a more chronic course in the United Kingdom trial performed by Russell *et al*. and the follow-up by Eisler *et al*.[70,72] These studies did not yield evidence that the specific type of family therapy administered was helpful for the older more chronic group.[65,70]

3.4. Psychosocial interventions based on addiction models Some programs attempt to blend features of addiction models, such as the 12 steps, with medical model programs which use cognitive-behavioral approaches.[73] However, no systematic data exist.

### Cochrane review of individual psychotherapy in the treatment of anorexia nervosa

A Cochrane review (which included seven small randomized control trials) of individual psychotherapy in the outpatient treatment of AN concluded that ‘treatment as usual’ may be less efficacious than a specific psychotherapy (CAT or FPT). No specific treatment was consistently superior to any other specific approach. One trial found a nonspecific therapy was favored over two specific psychotherapies (CBT and IPT).[74]

#### Long term outcome of treatment of anorexia nervosa

There are prospective cohort studies with comparison group, which have followed up patients of anorexia nervosa up to 10 years. At five-year follow-up,[75–77] approximately one-half of individuals in the Goteborg cohort with AN were considered recovered: 59% had no eating disorder diagnosis and 41% had a good outcome according to Morgan-Russell General scale criteria. However, 6% continued to have AN, 22% had BN, and 14% had EDNOS. The AN group was significantly less likely than the non-disease comparison group to be at normal body weight during the past 6 months. They also remained significantly more symptomatic on several measures such as dietary restriction, concern about body weight, worry about appearance, and Eating Attitudes Test (EAT) scores.

By 10 years,[78–80] the Morgan-Russell General scale outcomes had improved in the AN cohort. One-half had a good M-R scale outcome; the percentage with a poor outcome had declined from 24% at five years to 10% at 10 years. At follow-up, 27% had an eating disorder diagnosis.

### AN treatment: Predictors of response vs. predictors of non-response

Studies have found that patients who no longer had an eating disorder on follow-up were significantly less likely to be depressed or suffer from an anxiety disorder (except for obsessive compulsive disorder).[81] Significant predictors of chronic AN (intermediate or poor outcome) included a hostile attitude towards one’s family, extreme compulsive drive to exercise,[82] a history of poor social relationships preceding onset of illness,[82] worse evaluation scores concerning hypochondriasis, paranoia, and psychopathic deviance.[83]

Shorter duration of AN episode significantly predicted recovery after four years[84] and eight years.[85] In one study, relapse was greater among those whose duration of therapeutic contact was less than one year.[86] Higher percentage of average body weight (ABW) at intake predicted both a shorter time to both full and partial recovery.[87]

Defining recovery as a lack of symptoms for at least eight consecutive weeks, one study compared outcomes for restricting and binge/purge subtypes of AN. At four-year follow-up, full recovery was achieved in 17% of individuals with the AN binge/ purge subtype and in 8% of individuals with the AN restricting subtype. Rates for partial recovery were 81% in the binge/purge subtype and 54% in the restricting subtype.[84] After eight years, significant differences in recovery rates between subtypes were no longer observed.[88]

### Factors predictive of mortality in anorexia nervosa

In the UK study, predictors of death included weight less than 35 kg at presentation and more than one inpatient admission.[89] In another study that followed patients for approximately nine years, significant predictors of mortality included greater severity of alcohol use disorders, greater severity of substance use disorders, worse social adjustment, and worse Global Assessment of Functioning (GAF) scores.[90]

Suicide was a common cause of death. Among a group of US females with adolescent AN onset, the suicide-related SMR was 58.1, significantly higher than that for the population as a whole.[91] Thirty per cent of the patients had a history of suicide attempts before they entered the study; during the study, 22% attempted suicide. A history of suicide attempts at intake, greater drug use, participation in individual therapy, use of neuroleptic medications, and older age at disease ons *et al*. l predicted a first suicide attempt during the course of the study.[92]

## TREATMENT OF BULIMIA NERVOSA

### 1. Medications

#### 1.1. Antidepressants

Early observations that individuals with bulimia nervosa exhibit an elevated lifetime prevalence of mood disorders, together with an elevated prevalence of mood disorders, in their first-degree relatives, prompted initial trials of antidepressants for the acute treatment of bulimia nervosa.[93] In these trials, antidepressants appeared to be effective for bulimia nervosa regardless of whether or not the patient was clinically depressed.

##### 1.1.1. Selective Serotonin Reuptake Inhibitors

**Fluoxetine:** Six trials compared fluoxetine to placebo in outpatient and inpatient settings. Overall, fluoxetine (60 mg/day) administered for eight to16 weeks led to significant reduction in binge eating in most but not all studies.[94–99] Fluoxetine (60 mg/day) also performed significantly better than fluoxetine (20 mg/day) in decreasing binge eating.[95] No effect of Fluoxetine (60 mg/day) compared with placebo was observed in the one study in which patients were already receiving intensive inpatient psychotherapy.[98] Fluoxetine (60 mg/day) was superior to placebo in decreasing purging behavior, although not in the inpatient setting.[94–98] All six fluoxetine trials either failed to report abstinence rates or did not report whether abstinence rates differed significantly between drug and placebo groups.

With reference to eating-related attitudes, Fluoxetine (60 mg/day) was associated with significant improvements in measures of restraint, weight concern, and food preoccupation and with eating disorders inventory (EDI) subscale scores of bulimia, drive for thinness, and body dissatisfaction.[94–97] Fluoxetine had mixed results on depression and anxiety. Some studies showed greater efficacy than placebo in decreasing depression scores.[94–99]

**Fluvoxamine:** To compare maintenance of therapeutic gains and prevention of relapse of BN after inpatient treatment, Fichter *et al*. compared fluvoxamine (average dose 182 mg/day) with placebo for 19 weeks with medication started before discharge.[100] Patients treated with fluvoxamine reported fewer urges to binge, lower frequency of vomiting and lower depression scores than those receiving placebo. Both groups gained weight, with no differences between groups.

**Sertraline:** In a study of outpatients with BN by Milano *et al*. which compared sertraline (100 mg/d) with placebo, the group treated with sertraline had a statistically significant reduction in the number of binge eating crises and purging compared with the group who received placebo at 12 week endpoint.[101]

##### 1.1.2. Trazodone

In a six-week trial of trazodone (400 mg) versus placebo, trazodone led to significantly greater decrease in the frequency of binge eating and vomiting and in fear of eating.[102]

##### 1.1.3. Tricyclic Antidepressants

In a six-week trial, desipramine (200-300 mg/day) was significantly more effective than placebo in decreasing binge eating, vomiting, and scores on the eating attitudes test (EAT) and body shape questionnaire (BSQ).[103] Both self-reported depression and anxiety were significantly decreased in the desipramine group compared with the placebo group; clinician rated depression did not differ significantly.

##### 1.1.4. MAO inhibitors

**Brofaromine:** An eight-week trial of brofaromine (mean dose 175 mg/day) revealed no differences between the active drug and placebo on binge eating or psychological features of the eating disorder.[104] Brofaromine did lead to significant reductions in vomiting frequency.

#### Cochrane review of antidepressants in bulimia nervosa

A Cochrane review of 19 randomized controlled trials of antidepressants versus placebo in the treatment of BN found similar efficacy for different groups of drugs.[105] Use of a single antidepressant was clinically effective for the treatment of bulimia nervosa when compared to placebo. Patients treated with Tricyclic Antidepressants (TCAs) dropped out more frequently compared to placebo. The opposite was found with fluoxetine, suggesting it may be a more acceptable treatment. Independence b/w antidepressant and antibulimic effects could not be evaluated due to incomplete published data.

#### 1.2. 5-HT_3_ antagonist

**Ondansetron:** In a four-week trial of ondansetron versus placebo (self-administered upon urge to binge or vomit), the active drug led to significantly greater decreases than placebo in binge and vomit frequencies and time spent engaging in bulimic behaviors, and to significant increases in normal meals.[106]

#### 1.3. Other medications

A number of other medications have been used experimentally for BN without evidence of efficacy, including fenfluramine and lithium carbonate.[107,108] Fenfluramine has been taken off the market because of links between its use (mainly in combination with phentermine) and cardiac valvular abnormalities. Lithium may occasionally be used concurrently for the treatment of co-occurring conditions.

The opiate antagonist naltrexone has been studied in three randomized trials at dosages used for treating narcotic addiction and preventing relapse among alcohol-dependent patients (50-120 mg/day). The results consistently show that the medication is not superior to placebo in reducing bulimic symptoms.[109–111] In a small double-blind, crossover study involving higher dosages (e.g., 200-300 mg/day), naltrexone did appear to have some efficacy.

Topiramate, an antiepileptic agent, has been shown to be efficacious in treating BN symptoms in randomized trials.[112,113] Topiramate was found be efficacious in reducing uncontrolled eating, body dissatisfaction, dieting, food preoccupation, and anxious mood, and in increased patient-rated percent improved compared to placebo.

As in AN, carbamazepine and valproate may be effective in treating patients with bulimia nervosa also when they are used to treat an associated psychiatric (e.g. mood) or neurological (e.g. seizure) disorder.[59]

### 2. Psychosocial interventions

#### 2.1. Cognitive behavioral therapy (CBT)

CBT specifically directed at the eating disorder symptoms and underlying cognitions in patients with BN is the psychosocial intervention that has been most intensively studied in adults and for which there is the most evidence of efficacy.[114–126]

In comparison with individually administered CBT and IPT tailored for BN, CBT was associated with a significantly greater probability of remission than IPT and with greater decreases in vomiting and restraint and binge eating at the end of treatment.[115,127] In one study, at one-year follow-up, these differences were no longer apparent.[127] However, when administered in group format, both treatments led to significantly greater decreases than waiting list control on days binged, psychological features of the eating disorder, disinhibition, and restraint, although no differences were observed between CBT and IPT.[128] When compared directly, both group and individual administration of CBT showed decrease in objective and subjective binge episodes, vomiting, laxative use, over exercise and EDI bulimia, drive for thinness, and body dissatisfaction subscale scores.[129] Group CBT was associated with greater decreases in anxiety; individual CBT was associated with significantly higher rates of abstinence.

In studies that attempted to dismantle CBT to determine the "active" ingredients of this multimodal intervention, the cognitive component emerged as critical to therapeutic outcome. Complete CBT, including both cognitive and behavioral components, led to better eating-related outcomes than behavioral therapy components alone, to lower relapse rates than exposure with response prevention (ERP), and to greater abstinence than a self monitoring-only intervention.[127,130,131] ERP is a treatment in which patients are exposed to either high-risk binge or high-risk purge cues and the "response’"- either binge eating or purging is prevented until certain criteria are met. Two studies examined the additive efficacy of ERP to a core of cognitive or cognitive-behavioral therapy. Agras *et al*. found no additive benefit of ERP to CBT.[131] Similarly, Bulik *et al*. found no evidence of added efficacy when augmenting cognitive therapy with ERP.[121] In other comparisons, cognitive therapy performed better than support only; adding a cognitive component to nutritional counseling led to a significantly greater decrease in drive for thinness than nutritional therapy alone.[132] CBT was superior to nutritional counseling alone in improving core binge eating, vomiting, laxative use, and body dissatisfaction. CBT also led to significantly greater decreases than supportive-expressive therapy (a nondirective psycho dynamically oriented treatment) in EDI bulimia, EAT scores, food preoccupation, eating concerns, and depression.[133] Exercise therapy was superior to CBT at 18-month follow-up in improving drive for thinness, laxative abuse, and binge eating.[134]

It has been suggested that CBT might be adapted for children and adolescents with developmental considerations keeping in mind to make it more suitable for the management of eating disorders in this age group.[135]

#### 2.2. Dialectic behavior therapy (DBT)

Safer *et al*. compared DBT with waiting list control and found DBT superior in reducing the number of binge and purge episodes measured in the last four of 20 weeks of treatment.[136]

#### 2.3. Family therapy

Family therapy has been reported to be helpful in the treatment of BN in a large case series of adults, but more systematic studies are not available.[137]

#### 2.4. Self help trials

In a direct 18-week comparison of guided self help (manual including visits with non-specialists in eating disorders to check on progress) with group CBT, both treatments significantly decreased binge eating, vomiting, laxative use, EDI bulimia, drive for thinness, and body dissatisfaction.[138] At one-year follow-up, individuals in the self-help group showed greater reductions in vomiting and EDI bulimia. CBT was associated with greater reductions in drive for thinness over the treatment period and at follow-up. Both treatments significantly improved depression, with no differences between groups at the end of treatment; however, at one-year follow-up, individuals in the self-help group had lower depression scores. Of those who completed treatment, a significantly greater number of individuals in the self-help group than in the CBT group were in remission for more than two weeks at the end of treatment (74% vs. 44%). No significant change was seen in weight, although those in the self-help condition weighed significantly more at one year.

In addition, Carter *et al*. found that both CBT-based and nonspecific self-help approaches led to significant decreases in objective binge episodes and purging; the waiting list did not.[139] CBT-based self-help was associated with greater reductions in reducing intense exercise than nonspecific self help or waiting list.

Durand and King compared general practitioner (GP)-supported CBT-based self-help with specialist outpatient treatment.[140] The duration of treatment was at the clinician’s discretion. Patients in both groups reported significant decreases in depression and scores on the bulimic investigatory test Edinburgh (BITE) and eating disorders examination (EDE) total; however, binge eating and vomiting did not drop significantly. Dropout rates were similar across groups (24% in the GP group and 18% in specialist care).

A study by Thiels *et al*. compared 16 weeks of CBT only with guided self-change using a manual.[141] Guided self-change included 16 sessions with a therapist encouraging use of the manual and addressing motivation, obstacles, and emergent crises. Significant decreases occurred in overeating, vomiting, BITE scores, EAT scores, and depression for both groups combined. Only on BITE scores did the CBT group perform significantly better than the guided self-change group.

In a recent randomized controlled trial of CD-ROM based cognitive-behavior self-care for bulimia nervosa authors found that CD-ROM-based delivery of this intervention, without support from a clinician, may not be the best way of exploiting its benefits.[142]

#### 2.5. Support groups/12-step programs

Some patients have found ‘Overeaters Anonymous’ and similar groups to be helpful as adjuncts to initial treatments or for prevention of subsequent relapses, but no data from short or long-term outcome studies of these programs have been reported.[143,144]

## COCHRANE REVIEW OF PSYCHOTHERAPIES IN BN

A Cochrane review by Hay *et al*. which included 40 randomized controlled trials supported the efficacy of CBT, particularly CBT-BN, in the treatment of BN.[145] It found other psychotherapies to be efficacious also, particularly interpersonal therapy in the longer term. Exposure and response prevention did not appear to enhance the efficacy of CBT. The review concluded that psychotherapy alone is unlikely to reduce or change body wt in people with BN.

### 3. Medication plus psychosocial interventions

#### 3.1. Tricyclic antidepressant and CBT

One complex trial compared desipramine treatment of different durations with or without CBT (16 vs. 24 weeks) with CBT only.[146] The overall drop-out rate was 25%. The 16-week combined treatment was better than despiramine alone only for decreasing binge eating and purging. Longer combined treatment was significantly better than despiramine alone on binge eating, vomiting, dieting preoccupation, and hunger. Abstinence rates and weight change did not differ across groups. At one-year follow-up, the combined 24-week intervention and CBT alone were both better than the 16-week drug-only treatment in decreasing binge eating and vomiting. The 24-week combined treatment was also superior to 16-week drug-only treatment in decreasing binge frequency, dietary preoccupation, disinhibition, and hunger.[147]

#### 3.2. Selective serotonin reuptake inhibitor and CBT

Three trials used fluoxetine as the drug intervention. Comparing fluoxetine (60 mg/ day) to CBT only and to fluoxetine (60 mg/day) plus CBT in a 12-week trial, Goldbloom *et al*. used intention-to-treat analyses and found no difference across groups on eating-related measures.[148] In completers, all three interventions led to significant improvement in core bulimic symptoms; however, both combined treatment and CBT alone led to greater decreases than fluoxetine alone in objective and subjective binges and vomiting episodes. Abstinence rates, depression scores, and weight did not differ across groups. Dropout was highest in combined treatment (55%) versus fluoxetine (39%) and CBT only (35%).

Walsh *et al*. compared fluoxetine (60 mg/day) with placebo, each with or without self-help, in the form of a cognitive-behavioral self-help book.[149] Fluoxetine (either alone or with self-help) was associated with significantly decreased objective binge episodes, vomiting, restrained eating, and depression. The self-help book had no independent effect. No differences emerged on weight change. Dropout was high: 54% in fluoxetine plus guided self-help to 88% in placebo plus guided self-help.

Using the same design, Mitchell *et al*. found fluoxetine to be associated with a significantly greater decrease than placebo in vomiting episodes but not binge eating episodes.[150] No significant differences emerged in abstinence rates or depression and self-help had no independent effect. Dropout was low: none in fluoxetine only and fluoxetine plus self help, 5% in placebo only and placebo plus self-help.

#### 3.3. Multiple drugs and CBT

Walsh *et al*. examined supportive psychotherapy, CBT, both with or without placebo and with or without medication, and medication alone in a five-group 16-week comparison.[151,152] They started patients on desipramine (mean dose 188 mg/day) and switched nonresponders to fluoxetine (60 mg/day) after eight weeks. Analyses combining all arms of the study that included CBT versus all arms of the study that included supportive therapy indicated that CBT was superior to supportive therapy in reducing binge and vomit episode frequencies. Behavioral interventions plus medication were superior to behavioral interventions alone in reducing binge frequency, EAT scores, depressed mood, weight, and in increasing remission rate. CBT plus medication was superior to medication alone in reducing binge and vomit frequencies, EAT scores, body image, and increasing remission rate by self-report. Medication alone was superior to CBT alone in reducing BMI and weight. Medication alone was superior to supportive therapy plus medication in reducing binge and vomit frequency. Medication led to significantly greater decreases in depression scores. CBT was associated with greater likelihood of remission. The overall drop-out rate was 34 %.

Mitchell *et al*. randomized patients who did not respond to CBT to either interpersonal psychotherapy or fluoxetine (60 mg/day), which could be switched to desipramine in those who did not achieve abstinence.[153] No difference in abstinence was observed between the two groups. Overall, the sequential second-level treatment was associated with high dropout.

## COCHRANE REVIEW OF COMBINATION TREATMENT IN BULIMIA NERVOSA

A Cochrane review by Bacaltchuk *et al*. included five trials of antidepressants versus psychological treatment, five trials of antidepressants versus combination treatment and seven trials of psychological therapy versus combination treatment.[154] The review found 20% remission rate for single antidepressants compared to 39% for single psychotherapy. Remission rate of 42% was found for the combination vs. 23% for antidepressants. Thirty six percent remission rate was achieved through psychological approaches compared to 49% for the combination. However, statistically significant difference was noted only for combination treatments which were superior to single psychotherapy.

### 4. Additional interventions

#### 4.1. Guided imagery therapy

In a six-week controlled study involving 50 patients assigned to guided affective imagery therapy or a control group, those receiving the active treatment showed substantially more improvement in binge eating and purging behaviors and eating disorder-related attitudes.[155]

#### 4.2. Light therapy

In a small eight-week trial of 10,000 lux white light (active light) versus 50 lux red light (control), individuals in the active light group showed significantly greater decreases in binge eating than individuals in the control group.[156] Mood improved in both groups but no additional differences were observed for any other eating disorder, psychological, or biomarker outcome. The investigators did not provide long-term follow-up data. Given the size of this trial and the absence of follow-up, results should be viewed as preliminary.

#### 4.3. Crisis prevention

Individuals who were abstinent after a trial of CBT were randomized to either a crisis prevention group in which they were able to contact their clinician to receive up to eight additional visits over 17 months if they felt their condition was deteriorating or a control follow-up-only group.[157] The percentage of individuals who resumed binge eating and purging did not differ over the 17-month interval; however, none of the individuals in the crisis prevention group used any of their available calls despite the reappearance of bulimic symptoms.

#### Long term outcome of treatment of bulimia nervosa

There are case series studies which have attempted to see the long term outcome of bulimia nervosa. In one such study, patients who had received inpatient treatment for BN were followed for 12 years and compared with individuals who had never received treatment for an eating disorder on the Structured Inventory for Anorexic and Bulimic Syndromes, Expert-Rating version (SIABEX).[158] On this measure, at 12-year follow-up the BN group as a whole was significantly more symptomatic than the comparison group, including individuals with BN who were considered to be recovered. Among just the BN group, total EDI scores were worse at two years than at discharge but no different from discharge at six years.[159]

Case series studies with no comparison groups yielded mixed outcomes. At five years, in one study, BN patients across the age span recruited through general practitioners and psychiatrists had improved since baseline on a variety of measures including a significant reduction in recent mean number of objective bulimic episodes, self-induced vomiting episodes, and laxative misuse.[160–163]

In a US study of women who had sought treatment in Boston eating disorder programs, the percentage of the group that fully recovered increased over time. By seven years, 73% had achieved a full recovery (no symptoms or only residual symptoms for at least eight consecutive weeks) at some point during follow-up.[85] The trend was similar for partial recovery at some point during follow-up and included 98% of participants after seven years.[85]

#### Predictors of response vs. nonresponse in the treatment of bulimia nervosa

In the Fichter and Quadflieg study, lifetime psychiatric co morbidity predicted a significantly higher probability of having any eating disorder at two and six years but not at 12 years.[158] Significant predictors of continued BN (or AN) status at six years (adjusted for type of treatment and duration of follow-up) included paternal obesity and premorbid obesity.[161]

Prognostic factors significantly related to both categorical (full or partial remission vs. not in remission) and continuous outcomes (log of the number of months since last binge/purge episode), in a US study, included mood disorder, substance use and impulse control disorders.[164]

Baseline depression was both independent of and superior to bulimic symptoms in predicting body dissatisfaction at follow-up,[165] demonstrating a direct association between depression and body dissatisfaction, independent of bulimic symptoms. Recent prospective four-to-five-year follow-up studies also show that earlier age of detection of an eating disorder[166] and less co morbidity with personality disorders[167] are associated with better outcome.

## SUMMARY OF THE EVIDENCE

**Anorexia nervosa:** In acute stage of anorexia nervosa, weight restoration through nutritional rehabilitation should be the focus of treatment and nursing supervised oral refeeding of normal food for this purpose is recommended. Managing individuals with AN with medication only is inappropriate, based on evidence reviewed here. No pharmacological intervention for AN has a significant impact on weight gain or the psychological features of AN. Although mood may improve with tricyclic antidepressants, this outcome is not associated with improved weight gain.

For adult AN, we have tentative evidence that CBT reduces relapse risk for adults, after weight restoration has been accomplished. By contrast, we do not know whether the CBT approach is more helpful than others in the acutely underweight state, as one study found that a manual-based form of NSCM was more effective than CBT and IPT in terms of global outcomes during the acute phase. There are no replications of these studies. Family therapy, as currently practiced, has no supportive evidence for adults with AN.

**Bulimia nervosa:** Good evidence indicates that fluoxetine (60 mg/ day) reduces core bulimic symptoms of binge eating and purging and associated psychological features of the eating disorder in the short term. The 60 mg dose performs better than lower doses and may contribute to decreased relapse at one year. Preliminary evidence based on either single or a few trials exists for other second-generation antidepressants (trazodone and fluvoxamine), an anticonvulsant (topiramate), and a tricyclic antidepressant (desipramine). Replication for all of these medications is required.

Evidence for CBT in the treatment of BN is strong. Although IPT is also as effective, at one-year follow-up as CBT, based on one fair-rated study, symptomatic change appears to be more rapid with CBT.

Studies that combined drugs and behavioral interventions provide only preliminary evidence regarding the optimal combination of medication and psychotherapy or self-help. Although some preliminary evidence exists for incremental efficacy with combined treatment, given the variety of designs used and lack of replication, evidence remains weak.

## CRITIQUE AND FUTURE DIRECTION

The studies conducted in the management of AN, BN have a limitation of small sample size and short follow-up period. Across the studies, no effort has been made to study drug augmentation effects. The absence of trials combining medications and behavioral interventions is a serious deficit in the AN literature. The literature on AN has failed to distinguish sufficiently between interventions targeted at individuals before or after weight restoration and has failed to address the optimal approach to re nutrition. The AN literature is devoid of medication studies for adolescents; drug trials have focused exclusively on adults. Psychotherapies applied to eating disorders have been borrowed from other fields such as depression (CBT and IPT), anxiety disorders (exposure with response prevention) and personality disorders (DBT). Effort should be made to develop tailor-made behavioral interventions for eating disorders. Newer medications affecting hunger, satiety, and energy expenditure as well as commonly associated psychiatric symptoms and conditions need to be developed and tested. Further development and testing of professionally designed self-administered treatments by manuals and computer-based treatment programs would be useful.

Despite rigorous searching no study could be found, from this subcontinent, on the management of eating disorder. This could be because of rarity and atypical presentation of eating disorder in India. However, this should not limit us from searching for the cases with good clinical skills and conducting well designed treatment trials upon them to develop culture specific treatment modalities which in turn would benefit the patients.
